# Role of the Outer Membrane Protein OprD_2_ in Carbapenem-Resistance Mechanisms of *Pseudomonas aeruginosa*

**DOI:** 10.1371/journal.pone.0139995

**Published:** 2015-10-06

**Authors:** Jilu Shen, Yaping Pan, Yaping Fang

**Affiliations:** 1 Department of Laboratory Medicine, The First Affiliated Hospital of Anhui Medical University, Anhui Medical University, Hefei, Anhui, China; 2 Department of Laboratory Medicine, The Second Affiliated Hospital of Anhui Medical University, Anhui Medical University, Hefei, Anhui, China; University of Malaya, MALAYSIA

## Abstract

We investigated the relationship between the outer membrane protein OprD_2_ and carbapenem-resistance in 141 clinical isolates of *Pseudomonas aeruginosa* collected between January and December 2013 from the First Affiliated Hospital of Anhui Medical University in China. Agar dilution methods were employed to determine the minimum inhibitory concentration of meropenem (MEM) and imipenem (IMP) for *P*. *aeruginosa*. The gene encoding OprD_2_ was amplified from141 *P*. *aeruginosa* isolates and analyzed by PCR and DNA sequencing. Differences between the effects of IMP^R^ and IMP^S^ groups on the resistance of the *P*. *aeruginosa* were observed by SDS-poly acrylamide gel electrophoresis (SDS-PAGE). Three resistance types were classified in the 141 carbapenem-resistant *P*. *aeruginosa* (CRPA) isolates tested, namely IMP^R^MEM^R^ (66.7%), IMP^R^MEM^S^ (32.6%), and IMP^R^MEM^S^ (0.7%). DNA sequencing revealed significant diverse gene mutations in the OprD_2_-encoding gene in these strains. Thirty-four strains had large fragment deletions in the OprD_2_gene, in 6 strains the gene contained fragment inserts, and in 96 resistant strains, the gene featured small fragment deletions or multi-site mutations. Only 4 metallo-β-lactamase strains and 1 imipenem-sensitive (meropenem-resistant) strain showed a normal OprD_2_ gene. Using SDS-PAGE to detect the outer membrane protein in 16 CRPA isolates, it was found that 10 IMP^R^MEM^R^ strains and 5 IMP^R^MEM^S^ strains had lost the OprD_2_ protein, while the IMP^S^MEM^R^ strain contained a normal 46-kDa protein. In conclusion, mutation or loss of the OprD_2_-encoding gene caused the loss of OprD_2_, which further led to carbapenem-resistance of *P*. *aeruginosa*. Our findings provide insights into the mechanism of carbapenem resistance in *P*. *aeruginosa*.

## Introduction

*Pseudomonas aeruginosa* was ranked first among all antibiotic-resistant gram-negative strains according to the China National Antimicrobial Resistance Investigation Net annual report of 2011[1]. It was listed as one of the main pathogenic bacteria in nosocomial infection owing to its capacity to develop resistance to multiple classes of antimicrobials through intrinsic mechanisms and through the acquisition of transferable resistance determinants [2]. In spite of its resistance, *P*. *aeruginosa* continued to remain sensitive to carbapenems; imipenemis one of the most frequently used drugs for the treatment of *P*. *aeruginosa* infections in China. However, the accumulation of carbapenem-resistant mechanisms could probably further increase carbapenem MICs, leaving even fewer therapy options for clinicians against the multi-drug resistant *P*. *aeruginosa* [3]. Studies on the antimicrobial resistance in *P*. *aeruginosa* have been reported by the Chinese CHINET system; the resistance rates to imipenem and meropenem were 30.5% and 24.5% in 2008, 30.5% and 25.2% in 2009, and 30.8% and 25.8% in 2010, respectively [4]. Therefore, *P*. *aeruginosa* infections are now difficult to cure and have become life threatening.

OprD is a substrate-specific outer membrane porin of *P*. *aeruginosa*, which allows the diffusion of basic amino acids, small peptides, and imipenem into the cell [5]. For imipenem, OprD loss can push the MIC above the resistance breakpoint [6]. Most carbapenem-resistant *P*. *aeruginosa* strains are defective in expression of OprD[7]. Damien Fournier et al. have reported many types of mechanisms of CRPA, including the production of the spectrum β-lactamase or carbapenemases, over-expression of the efflux pump, and the loss of outer membrane porins, with porinOprD lost in 94(86.2%) of the strains in their study [8]. We believe that the loss of OprD may present the main mechanisms of carbapenem-resistance in *P*. *aeruginosa*.

In order to test this hypothesis regarding the contribution of the outer membrane protein OprD_2_to carbapenem-resistance in *P*. *aeruginosa*, the OprD_2_ genes were amplified and sequenced in the present study and the role of the protein in carbapenem resistance was analyzed.

## Materials and Methods

### Materials

#### Bacterial strains

We isolated 141 strains of *P*. *aeruginosa* with carbapenem-resistant (imipenem- or meropenem-bacteriostatic inhibition zones ≤13mm) from the First Affiliated Hospital of Anhui Medical University in China from January to December 2013. The quality control strain for the antimicrobial susceptibility test was ATCC 27853, and the strain PAO1 was employed as the standardized control for the analysis of the outer membrane protein OprD_2_ with SDS-PAGE electrophoresis.

#### Antibiotics

Fourteen antimicrobial agents were tested: imipenem (IMP), meropenem (MEM), gentamycin (GEN), amikacin (AMK), ciprofloxacin (CIP), levofloxacin (LVX), piperacillin (PIP), cefotaximes (CTX), cefepime (FEP), cefoperazone (CFP), cefoperazone/Sulbactam (CLS), piperacillin/tazobactam (TZP), aztreonam (ATM), and polymyxin B (POL). All antimicrobials were produced by OXOID (Britain). Imipenem was purchased from Hangzhou Merck Pharmaceutical Co., Ltd. Meropenem is a standard drug approved by the Ministry of Health Biological Products in China.

#### Medium and biochemical reagents

MH agar medium was purchased from OXOID. PCR detection reagents, buffers, Taqpolymerase, dNTPs, and the cloning Vector PMD18 carrier were obtained from Takara products (Dalian, China). Tris Base, glycine, acrylamide, N,N'-methylenebis-acrylamide, N,N,N',N'-tetramethylethylenediamine (TEMED), sodium dodecyl sulfate (SDS), ammonium persulphate (AP), and Coomassie Brilliant Blue R250 were provided by Sangon Biomart Co. (Shanghai, China). Low molecular weight standard proteins (Marker) were purchased from Shanghai Sibas biotechnology. Phosphate-buffered saline (PBS) and decoloring liquid were prepared in our laboratory.

#### Primers

The OprD primers (OprD-A: 5′-ATGAAAGTGATGAAGTGGAGCG–3′, OprD-B: 5′-TTACAGGATCGACAGCGGATAG–3′; fragment size: 1332bp) were designed according to the GenBank sequence (NC_002516) and synthesized by Shanghai Yingjun Biotechnology (Shanghai, China).

#### Bioinformatics software

DNAstar software (U.S. DNAstar) was used to visualize DNA bands, and Quality One gel imaging analysis software (Bio-Rad) was employed to visualize protein bands after SDS-PAGE.

### Methods

#### Antimicrobial susceptibility test

Susceptibility to 14 antibiotics was tested in all *P*. *aeruginosa* isolates using the K-B disk diffusion method following the guidelines of the Clinical and Laboratory Standards Institute (CLSI) [9]. In addition, the minimum inhibitory concentration (MIC) of imipenem and meropenem was determined using the agar dilution method. Imipenem and meropenem concentrations ranged from 0.06 to128 mg/L, and the bacterial inoculum size was 104 CFU/point. Susceptibility was interpreted according to CLSI breakpoints.

#### PCR amplification of the OprD_2_-encoding gene sequence

Yoneyama et al.[10] have reported two kinds of deletion mutations: one type of mutant had an 11bp deletion in the 395–405coding region, the other contained a 1024bp deletion from nucleotides -519 to 685 at the initiation codon. Primers detecting full-length OprD_2_ were designed in this study, and the OprD_2_ -encoding gene was amplified by PCR. PCR products were analyzed by agarose gel electrophoresis, and three possible results were expected: 1.) TheOprD_2_ gene features mutations or lacks a small fragment of 11bp. The gene products can amplify the corresponding bands are visible, but in order to distinguish between small deletions and mutations further sequencing analysis would be required. 2.) The OprD_2_ gene contains 1024-bp fragment deletions, the corresponding DNA segment cannot be amplified, and the electrophoresis results are negative. 3.) The OprD_2_ contains insertions, the primers can amplify the corresponding DNA fragments, and electrophoresis results are positive. The PCR amplification products were sequenced by Sangon Biomart Co. (Shanghai, China). Nucleotide sequences were analyzed and compared by BLAST.

#### Analysis of the outer membrane protein OprD_2_

There were 15 imipenem-resistant and 3 imipenem-sensitive *P*. *aeruginosa* (including the control strain PAO1) that contained large fragment deletions, missing small fragments, or inserted genes. Potentially resulting changes in the OprD_2_ outer membrane protein were analyzed in these strains using SDS-PAGE. The preparation of outer membrane proteins and SDS-PAGE were performed according to previously described methods[11,12]. After electrophoresis, the gel was stained with Coomassie Brilliant Blue R–250 for more than 4h, decolored for 4–8h, and then imaged using the Bio-Rad GelDoc XR Gel Imaging System. Quantity One image analysis software was employed to determine the relative content of protein bands of OprD_2_. Statistically significant differences between the IMP^S^ and IMP^R^ were analyzed with a *t*-test using the statistics software SPSS 13.0, with *P*<0.05 considered statistically significant.

## Results

### Antimicrobial susceptibility test

The susceptibility of the 141 *P*. *aeruginosa* strains to the 14 antimicrobial drugs tested is shown in Table 1. Three types of strains were categorized according to their resistance to imipenem and meropenem: IMP^R^MEM^R^ (94/141) was predominant and accounted for 66.7%, IMP^R^MEM^S^ accounted for 32.6% (46/141), and only a single IMP^S^MEM^R^ strain was detected and accounted for only 0.7% (1/141).

**Table 1:** Resistance of 141 *Pseudomonas aeruginosa* strains to 14 antimicrobial drugs, minimum inhibitory concentration (MIC, mg/L).

Antibacterial drugs	*Pseudomonas aeruginosa* (141 strains)					
	MIC range	MIC_50_	MIC_90_	sensitive(%)	intermediate(%)	resistance(%)
Imipenem	1–>128	16	32	0.7	0	99.3
Meropenem	2–>128	16	32	32.6	0	67.4
Gentamycin	0.25–>128	128	128	6.4	0	93.6
Amikacin	0.06–>128	64	128	45.4	0	54.6
Ciprofloxacin	0.125–>128	4	32	10.7	11.8	77.5
Levofloxacin	0.25–128	4	32	16.4	7.1	76.5
Piperacillin	0.06–>128	128	128	46.1	0	53.9
Cefotaximes	0.06–>128	128	128	22.7	16.3	61.0
Cefepime	0.06–>128	16	128	27.7	26.2	46.1
Cefoperazone	0.06–>128	128	128	4.9	14.2	80.9
Cefoperazone/sulbactam *	0.5–>128	64	128	17.0	22.7	60.3
Piperacillin/tazobactam *	2–>128	128	128	29.1	0	70.9
Aztreonam	1–>128	128	128	16.4	10.6	73.0
Polymyxin B	0.06–4	2	2	100	0	0

*Concentration of piperacillin and cefoperazone, respectively.

### PCR amplification of OprD_2_-encoding gene sequence

**Fig 1:**
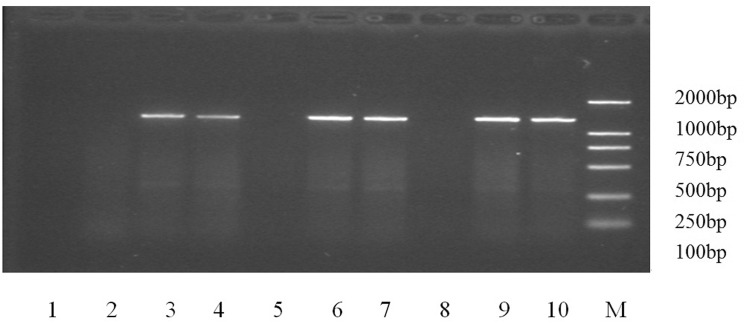
PCR amplification of the OprD_2_ gene shown for 10 selected *P*. *aeruginosa* strains. The electrophoresis results of strains 1, 2, 5, and 8 were negative, demonstrating that those strains had large fragment deletions. Strains 3, 4, 6, 7, 9, and 10 were positive, without any OprD_2_ gene mutations, lack of small fragments, or inserted fragments. M: Marker DL2000.

PCR amplification of the OprD_2_-encoding genes of the 141 CRPA strains showed negative gene amplification in 34 strains (Fig 1), proving that there were large fragment deletions of OprD_2_, while PCR amplification results were positive in the other 107 strains (Table 2). Six of the positive strains showed PCR amplification fragments larger than the expected 1332bp, and sequencing analysis revealed that the OprD_2_-encoding genes contained a fragment insert (IS 852bp). The amplified products of 96 IMP^R^ strains were missing small fragments in the OprD_2_-encoding gene sequence or contained multi-point mutations in different locations. The OprD_2_-encoding gene of 4 strains was identified to be encoding for VIM–2 type metallo-β-lactamases, and in the single 1 IMP^S^MEM^R^ strain nothing unusual was observed.

**Table 2:** OprD_2_ gene sequencing results of 141 CRPA strains.

Modification	No. of strains
Large segment missing	34
Inserted sequence	6
Small pieces lacking or multiple mutations	96
Normal sequence	5
Total	141

### Analysis of the outer membrane protein OprD_2_

**Fig 2:**
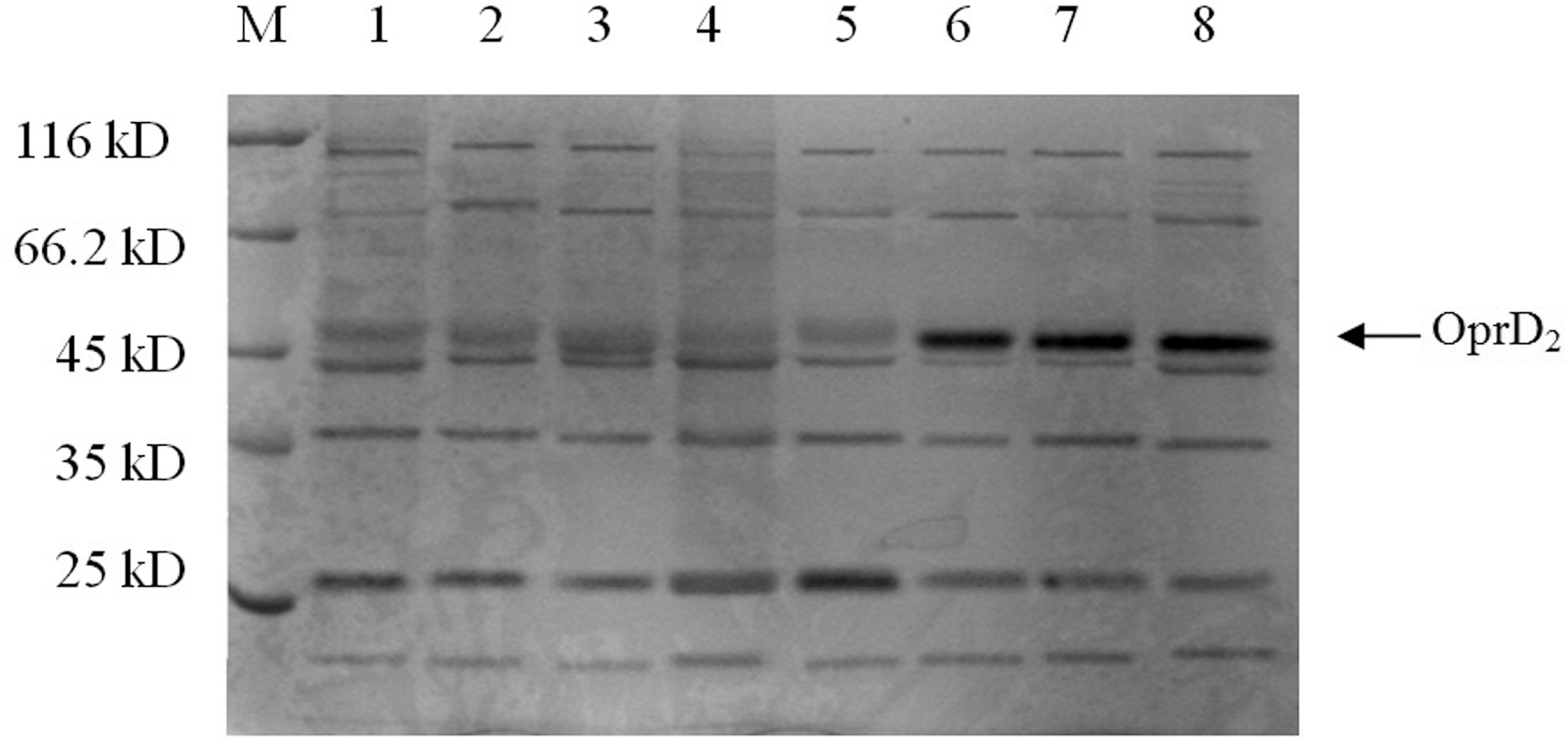
SDS-PAGE for the OprD_2_ protein in *Pseudomonas aeruginosa* strains. M = standard marker protein, lane 1–3 = IMP^R^MEM^R^ strains, lane 4–5 = IMP^R^MEM^S^ strains, lane 6 = IMP^S^MEM^R^ strains, lane 7 = for IMP^S^MEM^S^ strains, 8 = PAO1. The expected band size of 46 kDa was detected for the OprD_2_ protein in the two IMP^S^ strains and the PAO1 strain (6–8), while the size decreased in the IMP^R^ strains 1–5.

Changes in the outer membrane protein OprD_2_ were assessed by SDS-PAGE in 15 IMP^R^ *P*. *aeruginosa* strains with small or large fragment deletions or inserted genes and in 3 IMP^S^ *P*. *aeruginosa* strains (including the control strains PAO1). The 15 IMP^R^ strains showed an OprD_2_ protein of diminished size. In contrast, both IMP^S^ strains and the PAO1 control strain showed the expected OprD_2_ protein band at 46kDa (Fig 2). The protein bands for the analyzed 18 *P*. *aeruginosa* strains were quantified by gel imaging analysis (Table 3). The 15 IMP^R^ strains yielded an average quantitative result of 10.01, while the 3 IMP^S^ strains had a value of 17.17. Significant differences in the 46-kDa OprD_2_ protein between the two groups were determined with the sensitive mean *t-*test (*P* < 0.05).

**Table 3:** OprD_2_ outer membrane protein quantification in 18 *P*. *aeruginosa* strains.

	OprD_2_-encoding genetic strain * *						Drug-resistant phenotype	
Strains	Large fragment missing	Small fragments missing or multisite mutations	Inserted fragment	Normal fragment	OprD_2_ protein content *	mean	IMP	MEM
1	+	-	-	-	10.8	10.01^#^	R	R
2	-	+	-	-	10.2		R	R
3	-	+	-	-	9.8		R	R
4	-	+	-	-	9.6		R	R
5	-	+	-	-	10.3		R	R
6	-	+	-	-	10.5		R	R
7	+	-	-	-	8.9		R	R
8	-	+	-	-	9.1		R	R
9	+	-	-	-	9.5		R	R
10	-	+	-	-	10.2		R	R
11	-	+	-	-	11.1		R	S
12	-	+	-	-	10.5		R	S
13	-	+	-	-	10.1		R	S
14	-	+	-	-	9.8		R	S
15	-	+	-	-	9.8		R	S
16	-	-	-	+	17.5	17.17^##^	S	R
17	-	-	-	+	16.8		S	S
18	-	-	-	+	17.2		S	S

* Determined using Quantity One image analysis software.

* *+fragment missing or inserted, -normal sequence.

^#^ The mean value of strains 1∼15.

^##^ The mean value of strains 16–18.

## Discussion

The increasing prevalence of health care-associated infections caused by multidrug resistant *P*. *aeruginosa* (MDR-PA) is severely compromising the selection of appropriate treatments and is associated with high morbidity and mortality[13]. The 141 strains of *P*. *aeruginosa* analyzed in the present study were all strains with multiple drug resistance, with 19 (13.5%) of them being pan drug-resistant *P*. *aeruginosa* (PDR-PA[14]) strains. The availability of reliable antibiotics for patients infected with MDR-PA or PDR-PA is limited, especially for immunocompromised patients or those with underlying diseases. Drug resistance mechanisms should be elucidated and resistance surveillance needs to be strengthened in order to appropriately guide treatment choice and the clinical use of antibiotics.

OprD is a substrate-specific outer membrane porin of *P*. *aeruginosa*, and its loss can significantly reduce the susceptibility of the bacterium to carbapenems [15].Mutations in the OprD_2_-encoding genes lead to decreased expression or loss of the OprD_2_protein. The types of mutations are diverse and include multipoint mutations, gene deletion, frame shift mutations, and base replacement of large fragments[16]. Yoneyama et al. [10] reported two types of mutations in OprD_2_-encoding genes. The first type contains a small 11-bp deletion, causing a frameshift mutation and generating a premature termination that leads to an abnormal OprD_2_ peptide chain and the development of drug resistance. The other type of mutation was a large deletion encompassing the region from -519 to 685 (1024bp missing) across the promoter region; this mutant gene cannot be transcribed into mRNA, causing a loss of the OprD_2_ protein. The PCR amplification of the 141 clinical isolates in our study showed significant variation in the OprD_2_ gene, and the gene mutant site was different from the two deletion mutation types reported by Yoneyama. Negative PCR results were obtained for 34 strains, suggesting that the OprD_2_ gene in these strains had large fragment deletions. The other 107 strains were PCR-positive for the OprD_2_ gene, of which six strains contained an insert of 852bp, yielding PCR amplification of fragments larger than the expected 1332bp, which differed from previous results that had been obtained in our laboratory and in other studies that demonstrated OprD_2_ genetic variation with the absence or mutation of main fragment types. OprD_2_ gene expression in the four strains expressing metallo-β-lactamase genes and in the imipenem-sensitive (meropenem-resistant) strain was found to be normal. In the remaining 96 resistant strains, the OprD_2_- encoding gene sequence analysis revealed the lack of small fragments or multi-point mutation in different locations.

The outer membrane protein of *P*. *aeruginosa* is semi-permeable and can be considered as a molecular sieve that allows the passage of hydrophilic small molecular weight material [17]. Carbapenems as a class of small molecular weight hydrophilic β-lactam antibiotics can permeate through bacterial outer membrane porin proteins OprC, OprD_2_, and OprE, but OprD_2_ is the channel specific protein for imipenem[18]. For further study of the imipenem-resistance mechanisms in *P*. *aeruginosa*, we used SDS-PAGE of 18 *P*. *aeruginosa* outer membrane proteins. The results showed that the 2 IMP^S^ strains (of which one is the MEM^R^ strain) and the control strain PAO1 did not lack the outer membrane protein OprD_2_, while the 15 IMP^R^ strains exhibited a loss or decrease of OprD_2_. Gel imaging for further quantitative analysis showed that the level of the outer membrane protein OprD_2_ was significantly lower in drug-resistant strains than in the susceptible ones, with *t-* tests in both groups indicating statistically significant differences between the two groups (*P*< 0.05). These results suggest that a loss or reduction in OprD_2_ may be the primary mechanism for imipenem-resistance in *P*. *aeruginosa*. Analysis of the OprD_2_-encoding gene sequence identified large fragment deletions in 3 of the 15 strains with a loss or decrease of OprD_2_, while the remaining 12 strains featured missing or small fragments of multi-point mutations in different locations, suggesting that OprD_2_ gene deletion may be the molecular basis for the loss of OprD_2_.

To date, several types of carbapenem-resistance mechanisms have been reported, including efflux pump overexpression, production of metallo-β-lactamases, and mutational inactivation of the outer membrane protein OprD[19,20,21]. In this study, 96.5% (136/141) of the CRPA strains showed loss or insertion of the OprD_2_-encoding gene, resulting in the absence or reduction of OprD_2_ protein expression. Of the 141 CRPA strains analyzed, 99.3% were resistant to imipenem, and 33% were sensitive to meropenem. Among them was one IMP^S^MEM^R^ strain with no abnormal expression of OprD_2_, demonstrating that the OprD_2_ change in *P*. *aeruginosa* was the main reason for bacterial resistance to imipenem, which is consistent with the related literature[6].

In a previous study, the resistance of 141 CRPA strains to carbapenemases was studied, and only 4 strains were found to produce the metal-producing VIM–2 enzyme, as determined using the imipenem/EDTA combined disc test (MBL-CD) and the imipenem/EDTA Etest (MBL-Etest)[22]. However, no Oprd_2_ protein deficiency was detected in the 4 strains, which suggests that due to its ability to produce metallo-β-lactamase, *P*. *aeruginosa* can acquire resistance to the two drugs at the same time. However, the detection rate of metallo-β-lactamase was found to be very low. Therefore, the absence of the outer membrane protein OprD_2_ in *P*. *aeruginosa* may be the main reason for the carbapenem resistance.

In conclusion, 96.5% (136/141) of the 141 CRPA strains analyzed showed a loss or insertion of the OprD_2_ encoding gene, 4 strains produced metallo-β-lactamases, and in the IMP^S^MEM^R^ strain, the resistance mechanism was apparently not linked to OprD_2_ and was caused by another mechanism. In conclusion, the main mechanism for CRPA is putatively connected with the OprD_2_ protein; analysis of more CRPA isolates is required to confirm this association.
